# Flight rules for clinical AI: lessons from aviation for human-AI collaboration in medicine

**DOI:** 10.1038/s41746-026-02410-1

**Published:** 2026-01-31

**Authors:** Ariel Yuhan Ong, David A. Merle, Andreas Pollreisz, Siegfried K. Wagner, Mertcan Sevgi, Pearse A. Keane, Roman Huemer, Julian Oehling, Markus Jäger, Josef Huemer

**Affiliations:** 1 NIHR Moorfields Biomedical Research Centre, London, UK; 2 Institute of Ophthalmology, University College London, London, UK; 3 Oxford Eye Hospital, Oxford University Hospitals NHS Foundation Trust, Oxford, UK; 4 Center of Ophthalmology, University Hospital Tübingen, Tübingen, Germany; 5 Department of Ophthalmology and Optometry, Medical University of Vienna, Vienna, Austria; 6 Flight Safety Department, Lufthansa German Airlines, Frankfurt, Germany; 7 Department of Ophthalmology and Optometry, Kepler University Hospital, Linz, Austria

**Keywords:** Business and industry, Computational biology and bioinformatics, Health care, Mathematics and computing, Medical research, Scientific community, Social sciences

## Abstract

The parallels between medicine and aviation are well-recognised. The aviation industry’s early experience with automation improved safety and efficiency, but simultaneously introduced new vulnerabilities and occasionally created misplaced trust in complex systems. Aviation has developed a robust safety framework in response to these costly lessons. In this Perspective, which draws from the experiences of clinicians and aviation experts, we argue that it is now time for the medical community to consider how we can learn from these lessons as artificial intelligence (AI) becomes increasingly integrated into clinical care. We propose that this requires a shift in perspective from AI as “autopilot” to collaboration with a “digital copilot”, as well as considerations of practicalities such as scenario-based training, clinician benchmarking, and minimum unaided practice, with the ultimate aim of optimising human-AI collaboration to improve patient care.

The parallels between medicine and the aviation industry have long been recognised. Both involve high-stakes decision-making (often under uncertainty), and both emphasise a strong culture of safety and multidisciplinary team working to safeguard human life^1^. Given these similarities, medicine has borrowed much from the aviation industry over the past few decades: the origins of surgical checklists, safety time-outs, the “just culture” of incident reporting and analysis, and human factors simulation training can all be traced back to flight safety (Fig. 1)^1,2^, which continues to inspire other initiatives such as sustainable quality and safety improvement practices in healthcare^3^.

**Fig. 1:**
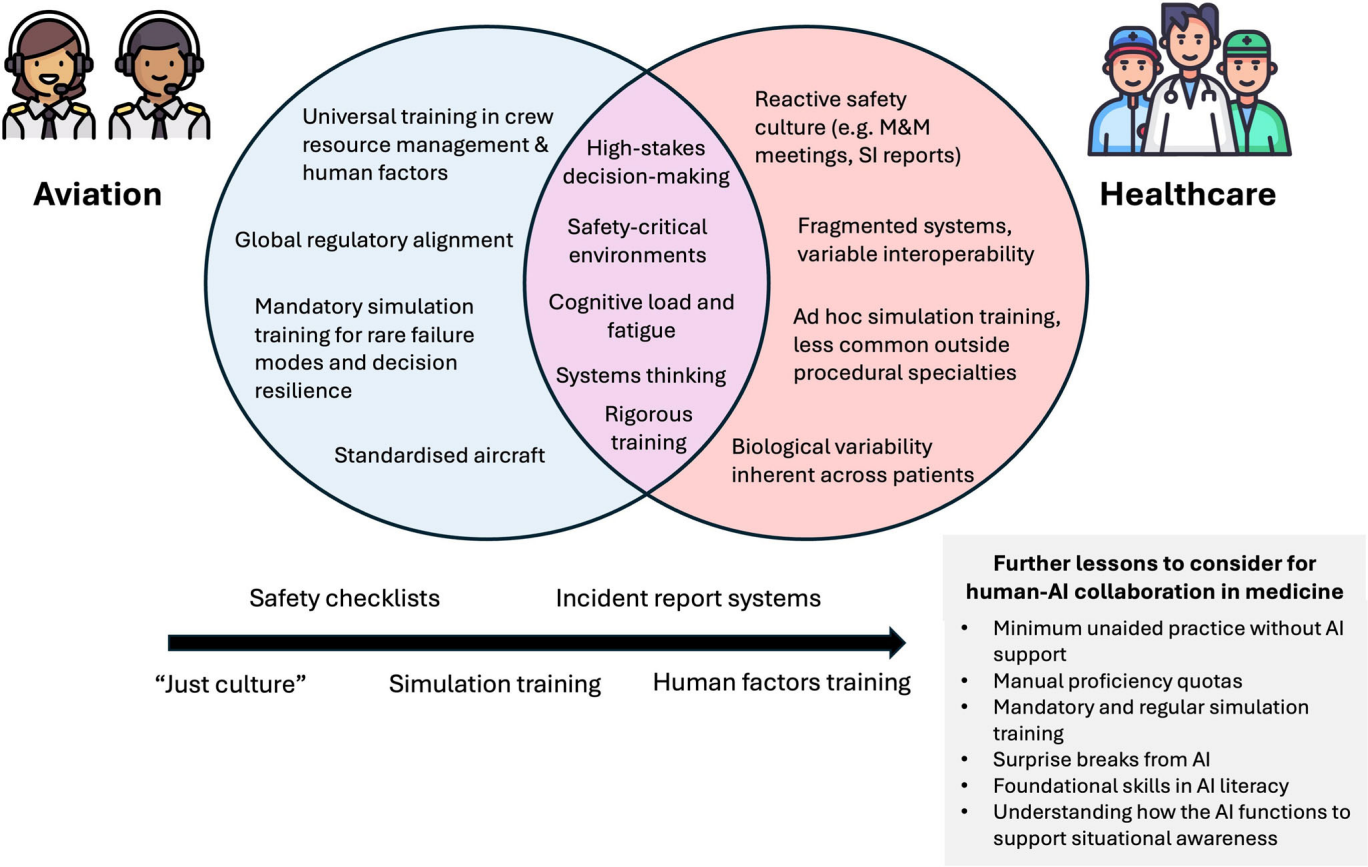
Conceptual relationship between the aviation and healthcare industries. The Venn diagram compares the similarities and differences. The directional arrow illustrates the historical adoption of key safety concepts such as safety checklists, simulation training, and “just culture” from aviation in healthcare thus far. The grey box highlights further lessons that healthcare can consider adopting from aviation, in order to aid human-AI collaboration. M&M morbidity and mortality, SI serious incident, AI artificial intelligence.

While significant quality gains have been achieved in healthcare over the same period of time, variability in practice and an appreciable morbidity and mortality from preventable medical errors persist^4,5^, even as airline passenger safety continues to improve at pace, with mortality rates halving every decade^6^. Part of this discrepancy might lie with contextual differences, such as the biological variability inherent across patients (as opposed to standardised aircraft), and patients being autonomous agents (as opposed to passive passengers on a flight). In addition, the field of aviation has well-resourced and central governance structures that support safety culture and accountability in aviation^7^, in contrast to healthcare, where analogous structures may be more fragmented or less well funded. However, despite some inherent dissimilarities, aviation’s track record demonstrates that meaningful progress is possible when safety is prioritised in a systematic and regulated manner.

These parallels have only deepened as artificial intelligence (AI) becomes increasingly integrated into clinical care. While the aviation industry’s early experience with automation improved safety and efficiency, it simultaneously introduced new vulnerabilities such as loss of manual skills, reduction of situational awareness, and misplaced trust in complex systems. Aviation has developed a robust safety framework around automation in response to these costly lessons. In this Perspective, we argue that with AI poised to profoundly reshape medical workflows, we must now consider how we can learn from these lessons to avoid making the same mistakes. Here, it is important to note that healthcare is not a single homogenous system, but comprises multiple different environments, settings, and clinical contexts^8^. Accordingly, the parallels drawn with aviation are not intended as a blueprint, but as a source of transferable principles that can be applied across different key areas where relevant.

## Early lessons from automation in aviation

Automation was introduced in aviation to improve safety and consistency. However, the “automation paradox” soon became apparent. This phenomenon describes how increasing automation erodes human skills and awareness, which begets the need for more automation, resulting in a vicious cycle that further increases the dependency on these systems^9^. Such behaviour led a senior pilot to coin the term “children of the magenta line”—a metaphor to describe younger pilots who became dependent on the use of autopilot and computer-generated ‘magenta lines’ displayed on their instruments, and who lacked the skills and confidence to fly the plane manually^9^.

Although accidents and the ensuing incident investigations catalysed a shift in aviation safety philosophy starting in the late 1970s, it took decades of incident investigations and persistent advocacy for policymakers and aircraft manufacturers to acknowledge that flight safety required investment in human capabilities and not just technology alone. The resulting reforms (spanning the late 1970s and 80s and becoming embedded in practice by the late 1990s) were grounded in the recognition that automation changes rather than eliminates human responsibility^10^. However, subsequent accidents, including the example of Asiana Airlines Flight 214 in San Francisco in 2013, demonstrates that this challenge persists even within relatively mature safety systems. In that incident, the pilots relied on the auto-throttle system to regulate engine thrust and airspeed, but failed to realise that they had misconfigured the automation. They fully relied on the automation to monitor the aircraft’s speed, which consequently became too slow to sustain flight, destroying the aircraft, killing four passengers and injuring many more.

Automation improves flight performance and reduces cognitive load, which is beneficial in the vast majority of situations where systems behave as expected. However, it can be catastrophic in rare instances of errors or unanticipated outcomes, especially as higher levels of automation comes at the cost of diminished situational awareness such as reduced attention to in-flight instruments^11^. De-skilled pilots, who are overly used to operating with automated systems, are often slower to detect anomalies, less prepared to take manual control, or more reliant on automated cues that may themselves be erroneous. Requiring pilots to actively measure and record key metrics such as altitude, fuel status, and time-to-destination and compare them to the pre-calculated flight plan at regular intervals are simple examples of actively seeking engagement to maintain an overview of the flight situation.

In medicine, there is the same risk that AI algorithms can enhance performance and efficiency or reduce workload under routine conditions but undermine clinicians’ vigilance, particularly when errors, system failures, or unexpected situations occur. Parallel examples are starting to emerge in clinical care—a recent study found that endoscopists who regularly performed AI-assisted colonoscopy performed worse at detecting adenomas—a 6.0 percentage point absolute reduction^12^, which has clinically significant implications for interval colorectal cancer risk^13^—after their AI assistant was removed from the equation, suggesting a potential dependency effect. Clinicians have also expressed other concerns about deskilling with AI exposure in terms of identifying clinical signs from physical examination^14^, even as inadequate clinical skills has been identified as a key cause of medical errors^15^. Instruction in the art of history-taking and examination has been central to the practice of medicine since the days of William Osler^16^, but increasing dependence on AI may accelerate the erosion of these skills as well as clinical reasoning—the iterative process of hypothesis generation, observation, and evidence synthesis at the heart of medicine^17,18^.

## From autopilot to digital copilot

Just as the introduction of automation changed the roles and responsibilities of pilots, the increasing integration of AI may also influence how clinicians practice medicine. Despite the growing tension between automation versus augmentation in medical AI^19^, in practice, very few algorithms truly function independently, as any AI health technology has to operate within the multidisciplinary and multilayered healthcare ecosystem. Varying degrees of human-AI teaming are required for different tasks and contexts, meaning that in reality, “autonomous” and “clinical decision support” algorithms are not dichotomous but are situated on a continuum.

Recognising this reframes AI not as an autopilot that replaces human input, but as a “digital copilot” that supports it. We argue that this conceptual shift provides a more useful model for medicine. Instead of passive oversight or a perfunctory “human-in-the-loop” design, clinicians and AI act as partners or collaborators, either as single human-AI units or in larger teams. Clinicians remain as the “pilot-in-command” and accountable for overall judgement, while the digital copilot can contribute consistency and precision. This is the basis of co-intelligence: a synergistic relationship that combines human contextual reasoning with algorithmic speed and pattern recognition^20^, with clinicians and AI systems operating as interdependent partners within a human-AI team^21^.

This framing also aligns with patient preferences—a large multinational survey found that patients consistently favoured a collaborative diagnostic approach led by clinicians when AI is involved in their care, and expressed reluctance for AI to reduce physician-patient interaction or replace human physicians altogether^22^. At the same time, the AI safety and ethics literature also cautions that accountability in AI-mediated care is inherently socio-technical and cannot rest with clinicians alone. Instead, clinical judgement should remain as a core component of dynamic approaches to safety assurance that extend beyond design-time safeguards to include developers, systems safety engineers, and organisations across real-world use^23^.

**Fig. 2:**
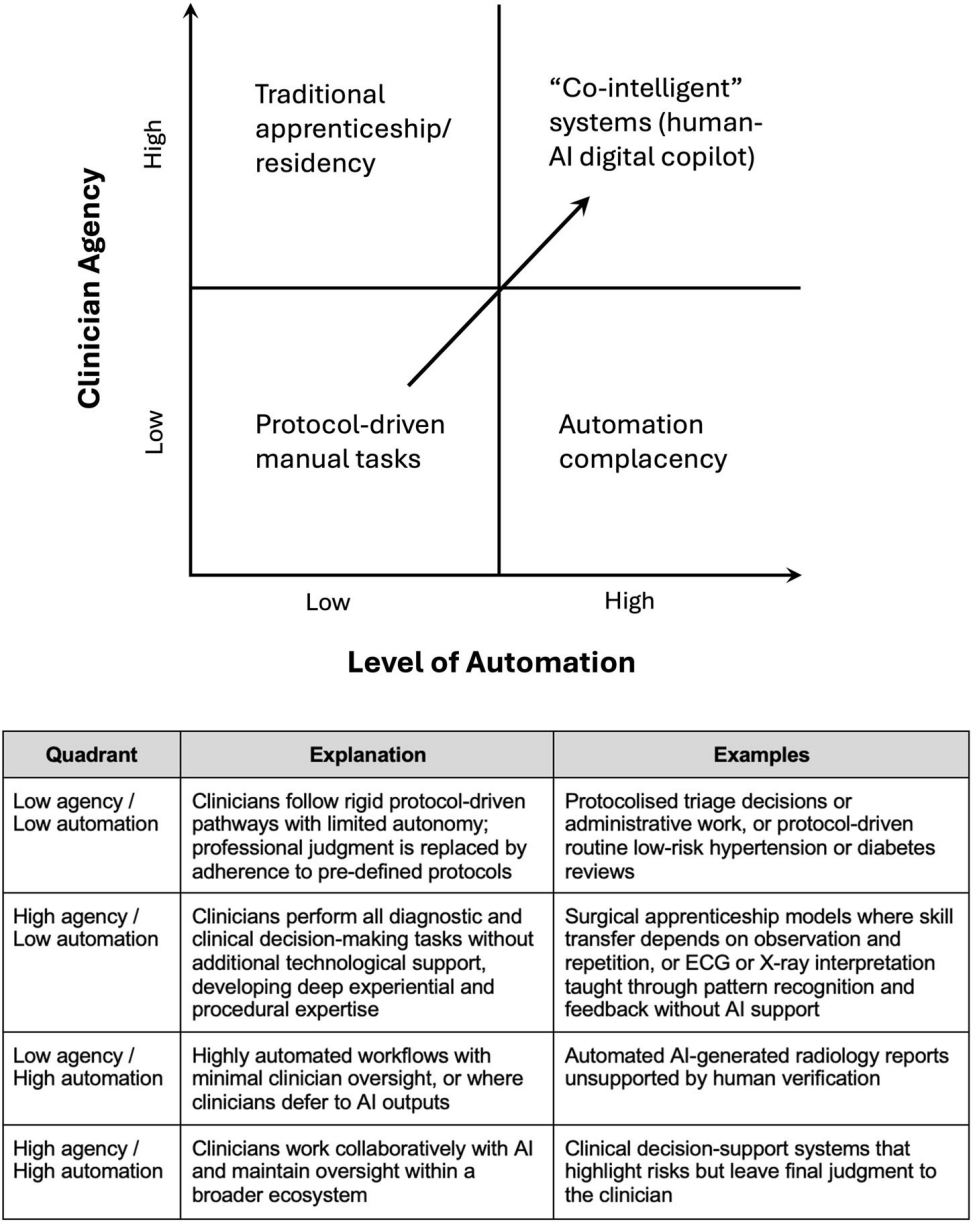
Matrix illustrating how clinician agency may interact with levels of automation in medical artificial intelligence, accompanied by an explanation of each quadrant. The bottom quadrants of the matrix represent the risk states of disempowered manual practice (low automation/ low agency) and automation complacency (high automation/ low agency). The upper quadrants represent more optimal configurations, from the traditional medical apprenticeship model (low automation/ high agency) to co-intelligent systems (high automation/ high agency), where clinicians maintain oversight. The arrow represents the trajectory towards co-intelligence and achieving automation efficiency without eroding human expertise.

This evolving relationship between humans and AI can be understood through the interplay between the level of automation and clinician agency, which draws from prior human factors research^24,25^ (Fig. 2). In low-automation/high-agency settings, clinical work resembles the traditional apprenticeship model. As automation increases, efficiency improves but clinicians may drift toward automation complacency and lose sight of situational awareness^24^. Conversely, rigid protocol-driven workflows can also diminish agency^26,27^. Both extremes limit clinicians’ ability to exercise their clinical judgement. We propose that the optimal configuration lies in high-automation/high-agency systems, where humans and AI function as co-intelligent partners. The challenge, then, is not to resist AI but to ensure that its adoption does not erode the very skills it seeks to support.

## The way forwards: translating aviation’s lessons into healthcare

Here, aviation offers another useful precedent—rather than striving to perfect automation alone, flight safety prioritises addressing human fallibility within increasingly automated systems^28^. This same principle is also applicable to medical AI (Fig. 1). We propose five key considerations to navigate these changes, inspired by lessons drawn from flight safety.

### Addressing the risk of skill erosion across generations by benchmarking clinicians and monitoring performance without AI

One of aviation’s most costly lessons is that skill decay secondary to automation is predictable and reversible, but only if this is actively addressed. To mitigate the risk of losing manual flight proficiency, pilots are actively encouraged to maintain their manual flying skills on routine flights under appropriate circumstances, for example when the workload is low or there is good weather. Similarly, as AI becomes more routine, minimum unaided practice without AI support should be considered for practicing clinicians. Ironically, while there is significant interest in benchmarking AI algorithms, the same level of enthusiasm has not applied to human clinicians, particularly in non-surgical specialties. The national diabetic retinopathy screening programme in the UK is one of the few examples where human graders are benchmarked on their diagnostic performance on a regular basis to improve quality and consistency^29^, but this is not done in routine clinical practice once clinicians have completed their specialty board exams (or equivalent). It is impossible to assess de-skilling without measurements. Institutions integrating AI (either “autonomous” or decision support systems) could consider monitoring human-AI concordance rates to assess for overreliance and complacency, as well as incorporating manual proficiency quotas, with performance on these no-AI cases providing a baseline benchmark for detecting drift for both the AI and the human user.

### Reconsidering how medical training is designed and structured to facilitate the development of fundamental cognitive and procedural skills before AI is introduced

For younger clinicians trained in an era where AI is increasingly ubiquitous and automating routine clinical tasks, the more fundamental concern is not “deskilling” but “never skilling” or “mis-skilling”^30^. A growing body of evidence suggests that learners develop shallower knowledge with AI-based tools as opposed to self-directed learning^31^. Addressing this risk requires intentional design in medical education, including careful rethinking of how clinical training is structured in an AI-rich environment. Early exposure should prioritise independent reasoning and experiential learning before automation is introduced, meaning that access to AI tools in clinical care should perhaps be restricted until basic clinical competency has been developed, thus allowing AI to function as a scaffold rather than a substitute for skill development. Alongside this, longitudinal monitoring of cognitive and procedural competence is needed to detect early signs of “never skilling” or “mis-skilling”, particularly in specialties where AI use is becoming increasingly routine. Trainees must remain active participants in diagnostic and clinical decision-making processes rather than passive recipients of AI outputs in order to avoid producing clinicians who are competent at using AI rather than understanding patients.

Ultimately, all of these issues are not unique to AI; similar debates have emerged with earlier waves of automation in other safety-critical industries^25^, as well as with previous generations of medical technologies^32^. The enduring tension lies in how to realise the benefits of these tools while helping doctors maintain agency, without eroding the human expertise on which these systems depend, as clinical judgement should remain the ultimate safety redundancy in AI-enabled practice. The rise of generative AI—particularly large language models (LLMs)—presents an additional challenge due to their occasional tendency to generate hallucinations, or plausible-sounding outputs that may not be fully grounded in factual evidence^33^. Confidently incorrect outputs may be misinterpreted as authoritative guidance, and justification chains by the model may not reflect valid reasoning. This stands in stark contrast to previous generations of safety-critical automation in aviation, which were designed with explicit failure modes and standardised alerts, which could signal when performance limitations are reached or exceeded. For example, autoland systems specify maximum allowable crosswind components and environmental conditions; pilots are prohibited from using such systems outside these certified parameters^34^. Whether and how generative AI can be engineered to exhibit comparable constraint, to communicate uncertainty, and to operate within clearly defined boundaries remains an open question that we need to consider as deployment in healthcare settings becomes more widespread.

### Defining foundational skills in AI literacy and the minimum digital and technical competencies that clinicians using AI should possess

To navigate these changes safely, medical curricula should also consider foundational skills in AI literacy, which some organisations are now beginning to articulate, albeit without significant consistency or traction as yet^35,36^. It is not enough to be able to operate an AI tool. Rather, clinicians should also be trained to use the technology appropriately and optimally^32^. To this end, understanding general concepts about AI they (such as LLMs being token predictors rather than an all-knowing oracle) is an essential starting point, as is an appreciation of AI limitations, failure modes, and susceptibility to bias, which has been acknowledged as a substantial concern given the risk of amplifying existing health inequalities if left unrecognised^37,38^.

In order to achieve this, there is a clear need for consensus around the minimum digital and technical competencies necessary for clinicians operating in AI-assisted clinical settings^39^. In addition, competency is not static but requires regular maintenance, especially given the rapid pace of AI advancement and the associated risk of obsolescence. As such, careful deliberation about how much and what to teach in medical school, postgraduate training, and in continuing professional development is also essential.

### Incorporating regular simulation-based training for human-AI teams to monitor dependence and instill situational awareness, and train clinicians when to trust and question their AI support

Robust training and simulation for human-AI teaming should also be considered. The aviation industry mandates line-oriented flight training^40^, where it is compulsory for pilots to spend a set number of hours a year in high-fidelity simulators to develop core competencies such as situational awareness. For example, the European Union mandates 48 h every 3 years, although the specific requirements depend on jurisdiction^41^. Evidence-based training for skill assessment and development rather than task completion has historically been the educational gold standard. This is a familiar concept in some fields of medicine such as ophthalmology, where “vitreous loss safety drills” have been introduced to help familiarise surgeons with the management of uncommon but potentially serious intraoperative complications, in order to equip them with the ability to handle them when they do arise^42^; however, this is often conducted on an ad hoc basis rather in a systematic or regulated manner. In addition, despite the increasing recognition that maintaining team-level situational awareness and shared mental models to develop high-functioning teams can improve patient management in other specialties such as anaesthesia^43^, this concept is uncommon in many other areas of medicine.

We propose going beyond this to consider scenario-based simulation training that recreate contexts where AI fails in order to monitor human dependence on AI systems, and to train clinicians when to trust, when to question, and when to override their “digital co-pilot”. In settings where AI use has become routine, introducing unannounced “surprise breaks” from AI can help assess readiness to operate safely without automation^44^. This should also incorporate structured debriefs where clinicians reflect on AI supported decisions in order to reinforce accountability and situational awareness, akin to the virtual waypoints required of pilots.

### Cultivating operational understanding of AI function in clinical practice to support safe engagement and disengagement

Finally, there is a need for clinicians to develop an operational understanding of how the AI systems they use function in clinical practice. For the average clinician, the goal is not to understand the inner workings of a neural network, but to be able to appreciate the rationale for its decision in operational terms (e.g. highlighting the factors that contributed to a risk score, or pixels in an image that contributed to its decision), in order to support safe engagement with AI outputs or oversight without overwhelming the user with extraneous detail. This ties in with the “explainability conundrum”, which captures a central tension between transparency and clinical utility. While there is significant interest in methods to develop explainable AI (XAI)^45^, overly detailed or poorly calibrated explanations can paradoxically worsen outcomes by leading to unwarranted trust and misinterpretation of model behaviour^45,46^. Explainability should perhaps be judged by its contribution to operational understanding, rather than by the depth or technical fidelity of the explanations alone.

This emphasis on operational understanding aligns with one of the “golden rules” for pilots, which is the need to understand the automated system’s function at all times. When that understanding is lost, the procedure is clear: to reduce the level of automation step by step, or to disengage it entirely, and to regain manual control until situational awareness is restored. A parallel can be drawn with closed-loop systems in anaesthesia, where clinicians must understand the operating limits and know when to override or switch to manual mode if patient parameters drift outside expected bounds^47^.

## Charting a new course towards human-AI collaboration in medicine

Aviation’s experience with automation shows that safety is not achieved by replacing humans in complex systems but by defining their role within them. Ultimately, learning from aviation should go beyond adopting simulation training or checklists alone—human-machine (or human-AI) interaction in medicine should be treated as a discipline in its own right, with defined competencies, measurable outcomes, and explicit recovery mechanisms when the AI fails. Accordingly, safety should be viewed as an emergent property of the human-AI team rather than the technology alone, and regulation will need to evolve accordingly. As such, beyond simply certifying AI as a medical device, as AI becomes more prevalent in healthcare, we should also consider how we regulate the human-AI dyad, and how we maintain competence, accountability, and situational awareness in this context. As we begin to deploy AI in healthcare, considering how we investment in governance and infrastructure will also be essential to avoid reproducing aviation’s early failures and the decades of work that have led to its contemporary safety systems. The future of medical AI will not be defined by how much we automate, but by how well we learn to collaborate in creating a synergistic partnership that augments rather than replaces human judgement, without ever losing sight of the ultimate aim of improving patient care.

## Supplementary information

Aviation_Manuscript_SupplementaryInformation

## Data Availability

No datasets were generated or analysed during the current study.
