# Supplementary material for: Flight rules for clinical AI: lessons from aviation for human-AI collaboration in medicine
Source: NPJ Digit Med. 2026 Jan 31;9:201. doi: 10.1038/s41746-026-02410-1 (PMC12963479; doi:10.1038/s41746-026-02410-1)
Supplement: Supplementary file 1 — Aviation_Manuscript_SupplementaryInformation [file 41746_2026_2410_MOESM1_ESM.pdf]

Supplementary S1: Glossary of key terms

Automation paradox: Increasing automation erodes human skills and awareness, which begets the need for more automation, resulting in a vicious cycle that further increases the dependency on these automated systems.

Deskilling: Loss of previously acquired manual, perceptual, or cognitive skills due to prolonged reliance on technology or automation, leading to degradation of independent competence over time.

Explainability conundrum: The tension between the desire for transparency in AI systems and the risk that excessively or poorly calibrated explanations may impede usability or encourage misplaced confidence in the AI system.

Human-AI teaming: A structured model of collaboration in which humans and AI systems operate as interdependent partners, melding human contextual reasoning with algorithmic speed and pattern recognition as a synergistic whole.

Misskilling: Development or reinforcement of incorrect habits or cognitive models through inappropriate interaction with automation, for example by learning to adopt flawed diagnostic heuristics.

Never skilling: Failure to acquire foundational skills because technology mediates or replaces the original learning process. ‘Never skilling’ is distinct from ‘deskilling’ in that the skill is never gained rather than gained and later lost.

Situational awareness: Dynamic awareness and understanding of environmental cues and system status that allows the individual to recognise what is happening, why it is happening, and what is likely to happen next. High situational awareness enables early detection of errors or unexpected events, whereas loss of situational awareness (e.g. due to overreliance on automation) can lead to medical errors.
